# Comparison of knowledge and confidence between medical students as leaders and followers in simulated resuscitation

**DOI:** 10.5116/ijme.5e01.f00c

**Published:** 2020-01-21

**Authors:** Veerapong Vattanavanit, Bodin Khwannimit, Thanapon Nilmoje

**Affiliations:** 1 Critical Care Medicine Unit, Division of Internal Medicine, Faculty of Medicine, Prince of Songkla University, Hat Yai, Songkhla, Thailand; 2 Cardiology Unit, Division of Internal Medicine, Faculty of Medicine, Prince of Songkla University, Hat Yai, Songkhla, Thailand

**Keywords:** Leader, follower, simulation, training

## Abstract

**Objectives:**

To compare both the knowledge and self-reported confidence levels between medical students as the team leaders and followers in shock resuscitation simulation training.

**Methods:**

A cross-sectional study was conducted with all fifth-year medical students participating in a shock resuscitation simulation-based training between May 2017 and March 2018. The simulation class was a 3-hour session that consisted of 4 shock type scenarios as well as a post-training debriefing. Medical students were assigned into groups of 4–5 members, in which they freely selected a leader, and the rest filled the roles of followers. Of 139 medical students, 32 students were leaders. A 10-question pre-test and post-test determined knowledge assessment. At the end of the class, the students completed a 5-point Likert scale confidence level evaluation questionnaire. A t-test was applied to compare knowledge scores and confidence levels between the leaders and followers.

**Results:**

At the end of the class, the knowledge scores between the leaders (M=6.72, SD=1.51) and followers (M=6.93, SD=1.26) were not different (t_(137)_= -0.81, p=0.42). In addition, the student confidence levels were also similar between the leaders (M=3.63, SD=0.55) and followers (M=3.41, SD=0.64) after training (t_(137)_=1.70, p=0.09).

**Conclusions:**

The knowledge and confidence levels were not different between either the leaders or followers in simulated resuscitation. With time-limit simulation training, we suggested every student may not need to fulfil the leadership role, but a well-designed course and constructive debriefing are recommended. Future studies should evaluate skills and longitudinal effects of the leader role.

## Introduction

Simulation-based medical education has matured, especially in anaesthesiology, emergency medicine, and critical care medicine regarding patient safety and resuscitation skills.^1^^-^^4^ Students gain many benefits, such as virtual reality experiences, no harm, and participation in a student-centred activity. A study revealed that simulation-based training helped students better understand shock resuscitation compared to a case-based discussion.^5^ Leaders gained more knowledge and skills in solving emergency problems compared to problem-based learning.^6^

A circulatory shock is a generalized form of acute circulatory failure associated with cellular dysfunction that is life-threatening and results in a high mortality rate.^7^ Rapid detection and prompt resuscitation are crucial to save the organs and lives of patients. Resuscitation skills are required for medical students in clinical clerkship. Education intervention utilizing simulation to practice shock resuscitation that is given to all medical students prior to graduation may help achieve the goal of taking care of patients in shock on the first day of residency.

Our institution provides simulation-based training in shock resuscitation for fifth-year medical students. In our previous study, medical students improved their knowledge and confidence levels in septic shock resuscitation.^8^ We have extended the simulation course in common types of circulatory shock resuscitation. However, due to time limitations in the group assignments, only one student has the opportunity to take the role of leader.

Growing evidence shows the importance of non-technical skills. For example, effective team leadership in resuscitation is a contributing factor in the effectiveness of resuscitation. The absence of leadership and poor teamwork was shown to be associated with poor cardiopulmonary resuscitation (CPR) performance and negative clinical outcomes.^9^^-^^14^ However, much evidence has focused on CPR and the overall skill of a team. A recent study reported paediatric residents assigned as leaders had significant greater perceived self-confidence in CPR compared with those who assumed the role of followers.^15^ To the best of our knowledge, no study has demonstrated the influence of the role of leader on the confidence levels of medical students in simulated shock resuscitation.

We aimed to explore the knowledge and confidence levels between medical students as leaders and followers after the completion of simulation-based shock resuscitation courses. We hypothesized that the role of a leader does not affect the knowledge and confidence levels and that every student needs to play the role of the leader in our course.

## Methods

### Study design and setting

This was a cross-sectional study conducted at a university-based medical simulation centre in the Faculty of Medicine at Prince of Songkla University, Thailand. The centre consists of several simulation labs with infant, paediatric, and adult patient simulators; a skills lab; computer-based simulators; multimedia debriefing room; and high-fidelity medical manikins. The simulation system includes simulation software programs for manikin control and respiratory and haemodynamic monitoring. Simulation sessions are digitally recorded for playback and debriefing.

### Study participants

All fifth-year medical students were invited to participate in the resuscitation course from May 2017 to March 2018 during their rotation through internal medicine. There were 8 rotations with approximately 18 students per rotation. A total of 139 medical students were enrolled; 32 students (23%) were leaders. Baseline characteristics of the medical students are shown in Table 1. A study plan and course objectives were provided for the participants. Our objectives for simulated shock resuscitation were the diagnosis of the types of shock, the cause(s) of shock, and giving initial management.

The Ethics Committee of the Faculty of Medicine, Prince of Songkla University, approved the study. A waiver of written informed consent was granted by the Ethics Committee at the Faculty of Medicine, Prince of Songkla University because it was a cross-sectional study involving existing curriculum in an educational setting. The study was conducted according to the Belmont report ethical considerations: all participants data were confidential, no harm would be afflicted upon participants during the study, and their refusal in doing tests or questionnaires in the study would have no impact on their course assessment or grades.

**Table 1:** Baseline characteristics, knowledge scores, confidence levels, and satisfaction between leaders and followers

Variable	Leaders n = 32	Followers n = 107	p-value
	Mean (SD)	Mean (SD)	
Male, n (%)	18 (56.2)	52 (48.6)	0.45
Grade point average^*^	3.26 (0.26)	3.18 (0.26)	0.13
Pre-test score^†^	5.47 (1.68)	5.27 (1.94)	0.60
Post-test score^†^	6.72 (1.51)	6.93 (1.26)	0.42
Pre-training confidence levels^‡^	2.38 (0.79)	2.39 (0.79)	0.91
Post-training confidence levels^‡^	3.63 (0.55)	3.41 (0.64)	0.09
Overall satisfaction^¶^	8.75 (1.29)	8.57 (1.20)	0.49

An independent sample t-test is used to compare means of two groups *Grade point average at fourth year in medical curriculum †Pre-test and post-test scores have maximum scores of 10 points ‡Confidence level: 1 (not at all) to 5 (very confident) ¶Overall satisfaction has a maximum score of 10 points (boring to fun)

### Shock resuscitation simulation course

All students had already passed a 1-hour didactic lecture on shock resuscitation in the fourth year of the medical curriculum. A 3-hour simulated shock resuscitation course was arranged for all students. The course began with an introduction of the goals and objectives. Students did a pre-test for 10 minutes. Then, teachers provided orientation to the manikins and assigned students into 4 groups corresponding to 4 types of shock resuscitation scenarios. Students freely selected their group and chose one leader; the rest of the students were followers. The resuscitation time consumed 10 minutes, and a 20-minute debriefing for each group was completed after the course. At the end of the class, students did the post-test, self-reported their confidence and gave feedback.

**Table 2:** Overall knowledge scores and confidence level between pre- and post-simulation training

Variable	Pre-training	Post-training	p-value
	Mean (SD)	Mean (SD)	
Overall (n=139)			
Test scores^ *^	5.32 (1.88)	6.88 (1.32)	< 0.001
Confidence levels^ †^	2.39 (0.78)	3.46 (0.63)	< 0.001
Leaders (n= 32)			
Test score^ *^	5.47 (1.68)	6.72 (1.51)	< 0.001
Confidence levels^ †^	2.38 (0.79)	3.63 (0.55)	< 0.001
Followers (n=107)			
Test score^ *^	5.27 (1.94)	6.93 (1.26)	< 0.001
Confidence levels^ †^	2.39 (0.79)	3.41 (0.64)	< 0.001

A paired sample t-test is used to compare means of two groups *Test scores have a maximum score of 10 points †Confidence level: 1 (not at all) to 5 (very confident)

### Shock patient simulation

The Laerdal SimMan high-fidelity patient simulator (Laerdal Medical, Stavanger, Norway) represents a realistic patient in different types of shock. Computer-controlled connections with the manikins showed the haemodynamic and respiratory parameters on a monitor.

Shock scenarios consisted of 4 common types of circulatory shock: septic, cardiogenic, obstructive and anaphylactic shock (an example scenario can be found in Appendix 1). The authors wrote case scenarios for the 4 shock types. To validate the scenario, two experts received the scenario prior to student participation. They were interviewed and their feedback was used to improve the simulation.

Participants were grouped into four teams for the resuscitation course. Each team consisted of 4–5 members. Students freely selected one student to be a leader. The team members played the roles of leader, nurses, and proceduralist(s). The course instructors assumed the roles of a family member, paramedic, consultant, and lab technician, as needed. Two instructors were present in the room to evaluate team performance and another instructor was in the computer control room.

### Debriefing

At the end of each shock scenario, instructors gave an immediate post-action reflection and feedback or debriefing. The debriefing used a standard format,^16^ including a reaction phase for each participant, followed by the advocacy inquiry approach^17^ to recognize participant frames, and lastly, the generalization and application of the experience to further patient care. Team performance in crisis resource management was also addressed during the debriefing.

### Assessment and survey

The students completed a pre-test at the beginning of the simulation course and a post-test at the end of the course. The tests consisted of 10 multiple choice questions to test their knowledge of the four types of shock resuscitation. The validity of the content evaluated by the three subject experts on each item of the tests was greater than 0.6, indicating the tests congruence. Thirty sixth-year medical students were the pilot group for test reliability. Cronbach’s alpha correlation technique was used to ascertain the reliability of the tests, which was 0.81. Task performance was evaluated by two simulation instructors using checklists. At the end of the course, the participants were given a survey questionnaire regarding their attitudes and confidence levels of the simulation course. The confidence levels utilized a 5-point Likert scale that ranged from 1 (not at all) to 5 (very confident).^8^ Participants responded to the tests and questionnaires via Google Forms. Our teaching assistants collected data.

### Data analysis

Descriptive statistics were generated from the test scores and attitude scales. Data were presented as mean and standard deviation (SD). Comparisons between the pre- and post-test scores and pre- and post-course confidence levels were analysed using the independent-samples t-test or paired sample t-test with significance set at a p-value less than 0.05. All statistical analyses were performed using R.

## Results

All participants completed the tests and questionnaires. The post-test scores between the leaders (M=6.72, SD=1.51) and followers (M=6.93, SD=1.26) were not different (t_(137)_= -0.81, p=0.42). The student confidence level as leaders (M=3.63, SD=0.55) was higher than that of the followers (M=3.41, SD=0.64) but not statistically significant (t_(137)_=1.70, p=0.09). The overall satisfaction in the shock simulation training was high in both groups (Table 1).

Overall, the knowledge scores of the medical students improved significantly (t_(138)_=10.55, p<0.001) along with the confidence levels (t_(138)_=13.83, p<0.001) after training. Both leaders and followers improved test scores and confidence levels after the simulation course (Table 2).

## Discussion

From our prospective observational study, students taking the role as the leader in our simulation-based training in shock resuscitation course did not gain more knowledge or confidence as compared with the followers.

Simulation-based training creates a safe environment for learning; however, this training requires significant costs and is time-consuming. Previous studies in the simulation of shock management training used various course durations from 5–7 hours^5^^,^^18^ to 1.5 days.^19^ These studies did not indicate whether every learner had the opportunity to be a leader.

Leadership is a non-technical skill and is an interpersonal skill that is vital and has a significant effect on patient safety outcomes.^20^^,^^21^ The importance of the role of the leader in simulation-based training has been studied. Incorporating team leadership as the main topic of CPR showed positive effects on its performance. Separate team leader training had an impact on communication skills and guideline adherence in CPR training.^22^ Students reported higher mental strain and concentration as a leader than they did in the role of follower.^23^

Not many studies have focused on leadership skill in shock resuscitation simulation training. Nguyen and colleagues^18^ reported an effective 5-hour course, including lecture, skill workshops, and a simulated case scenario in septic shock for medical students. The team consisted of 3-4 medical students who played the role of leader, nurse, and proceduralist(s). However, the confidence level of the leader was not stated. A study on serial simulation in the management of paediatric septic shock for residents improved the performance scores but did not mention leadership skills or team performance in the debriefing.^24^

During emergency situations, an accurate diagnosis and prompt management are crucial. Self-confidence is an important skill in effective decision making.^25^ Simulation training was shown to be an effective learning method to improve the confidence levels among medical students.^8^^, ^^26^ However, there is a lack of studies that reported whether the self-confidence came from the simulation training per se or came from playing the role of the leader in simulated shock resuscitation.

Our results revealed no difference in the confidence levels between the leaders and followers, with some reasons. First, the groups were small. Everyone had a different role to play but had a chance to express their opinions on the dynamics of the team. Second, constructive debriefing at the end of the simulation provided feedback from everyone concerning their skills and knowledge. Post-simulation debriefing improved the confidence and provided effective learning in the students.^27^

Our study had several limitations. First, we had a relatively small sample size, because we compared participants in the same level and academic year. The number of leaders was less than the number of followers, which may not show different effects. Second, simulation training was conducted in a single centre. Therefore, generalizability should be a concern. Third, we did not survey the personal qualities and behavioural characteristics of the leaders. To understand leadership in detail, there are at least two ways of thinking: styles and situations.^28^

## Conclusions

In the setting of our shock simulated training, the role of leader did not affect the knowledge and confidence levels of the medical students during shock resuscitation. Due to time limitations, not every student was able to fulfil the leadership role. A well-designed course and constructive debriefing are recommended in order to improve the confidence levels of medical students. Future studies should assess skills and evaluate the longitudinal effects of the leader role.

### Acknowledgements

The authors gratefully acknowledge Mr Glenn Shingledecker in the International Affairs Department, Faculty of Medicine, Prince of Songkla University for the language correction services.

### Conflicts of Interests

The authors declare that they have no conflicts of interest.

## Supplementary material

Supplementary file 1 Appendix 1. Example of shock scenario: Septic shock
