# Supplementary material for: Comparison of knowledge and confidence between medical students as leaders and followers in simulated resuscitation
Source: Int J Med Educ. 2020 Jan 21;11:19–24. doi: 10.5116/ijme.5e01.f00c (PMC7246126; doi:10.5116/ijme.5e01.f00c)

Appendix 1.

Example of shock scenario: Septic shock

A 60-year-old male with a history of hypertension and diabetes mellitus.

- His chief complaint is a productive cough for two days associated with shortness of breath, fever and malaise. He reports no other symptoms.
- Estimated body weight is 60 kg.
- Initial vital signs: blood pressure (BP) 80/50 mmHg, heart rate (HR) 110/min, respiratory rate (RR) 30/min, SpO_2 85%, and body temperature (BT) 40 °C.

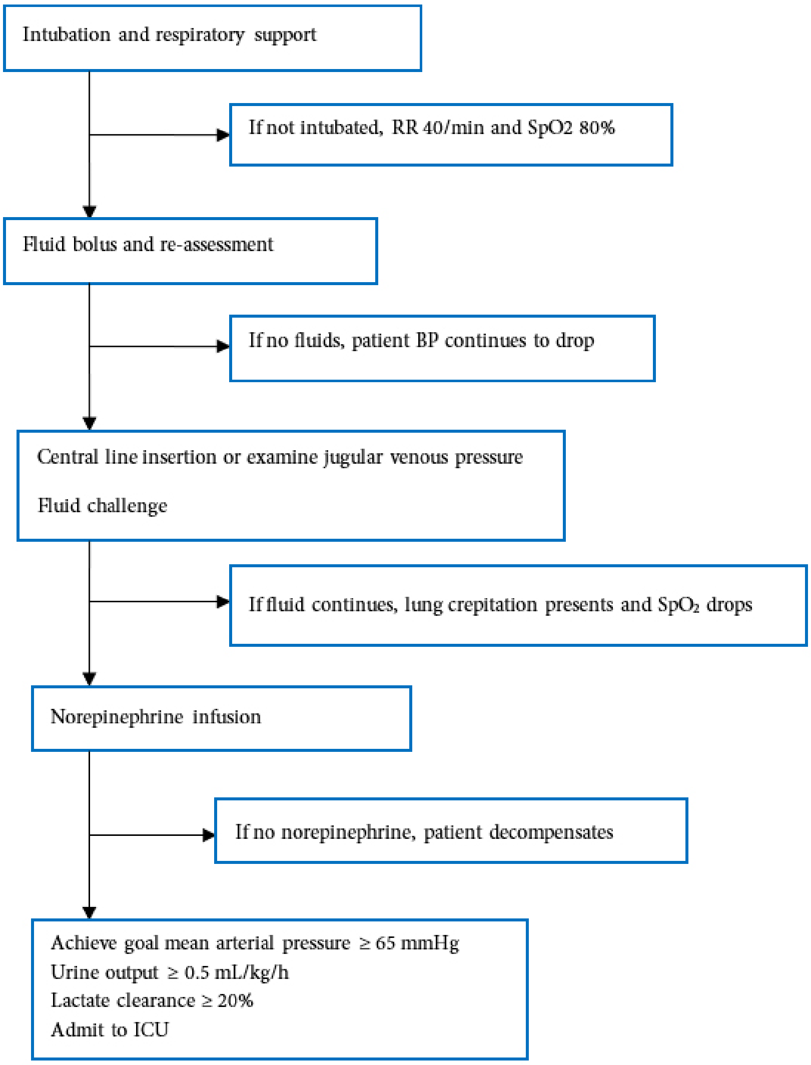

Supplement: Supplementary file 1 — Appendix 1. Example of shock scenario: Septic shock [file ijme-11-19-S1.pdf]
